# Brain Iron Metabolism, Redox Balance and Neurological Diseases

**DOI:** 10.3390/antiox12061289

**Published:** 2023-06-16

**Authors:** Guofen Gao, Linhao You, Jianhua Zhang, Yan-Zhong Chang, Peng Yu

**Affiliations:** Ministry of Education Key Laboratory of Molecular and Cellular Biology, The Key Laboratory of Animal Physiology, Biochemistry and Molecular Biology of Hebei Province, College of Life Sciences, Hebei Normal University, No. 20 Nan’erhuan Eastern Road, Shijiazhuang 050024, China

**Keywords:** oxidative stress, Parkinson’s disease, Alzheimer’s disease, stroke, neurodevelopment, iron chelator

## Abstract

The incidence of neurological diseases, such as Parkinson’s disease, Alzheimer’s disease and stroke, is increasing. An increasing number of studies have correlated these diseases with brain iron overload and the resulting oxidative damage. Brain iron deficiency has also been closely linked to neurodevelopment. These neurological disorders seriously affect the physical and mental health of patients and bring heavy economic burdens to families and society. Therefore, it is important to maintain brain iron homeostasis and to understand the mechanism of brain iron disorders affecting reactive oxygen species (ROS) balance, resulting in neural damage, cell death and, ultimately, leading to the development of disease. Evidence has shown that many therapies targeting brain iron and ROS imbalances have good preventive and therapeutic effects on neurological diseases. This review highlights the molecular mechanisms, pathogenesis and treatment strategies of brain iron metabolism disorders in neurological diseases.

## 1. Introduction

Iron is important for the physiology of the brain, participating in oxygen transport, energy production and the synthesis of DNA, myelin and neurotransmitters [1]. Brain iron deficiency (ID) impairs the development of neurons and glial cells, leading to abnormal neurodevelopment, which causes mental incapacity [2,3] and is correlated with neuropsychiatric disorders, such as depression and anxiety [4,5,6,7], while sufficient iron is crucial for ferrodifferentiation [8]. On the other hand, iron overload in the brain exacerbates the development of neurological diseases, such as Alzheimer’s disease (AD), Parkinson’s disease (PD) and stroke, because too much iron generates reactive oxygen species (ROS) that can destroy the cell membrane and induce cell death [9,10]. The incidence of neurological diseases has become increasingly higher with the advent of an aging society. Therefore, it is critical to maintain iron homeostasis in the brain and to investigate the underlying regulation mechanisms, which might provide better strategies to prevent and cure neurological diseases.

Recently, it has become much clearer how circulatory iron is absorbed into the brain parenchymal tissue across the blood–brain barrier (BBB), where hepcidin and ceruloplasmin (CP) regulate the iron transport process coordinately with ferroportin 1 (FPN1) [11,12,13,14]. In particular, neurodegeneration with brain iron accumulation (NBIA) is a set of rare monogenetic neurodegenerative diseases, which is characterized by iron accumulation in basal ganglia and other related brain regions [15,16]. Targeting iron transport and the regulation pathway to restore iron homeostasis has been applied in the prevention and treatment of NIBA, stroke and other neurological diseases. Iron chelation therapy with deferoxamine (DFO), deferasirox (DFX) and deferiprone (DFP) has been used in the clinic and for animal research [17,18,19,20,21,22]. As the therapeutic effects of iron chelators are not ideal in the treatment of iron-overload diseases, it is crucial to elaborate the detailed regulatory mechanisms of brain iron metabolism in these diseases to discover new targets and new therapeutic strategies for these neurological diseases. Therefore, in this review, we discuss the molecular mechanisms of brain iron metabolism, pathogenesis of iron-dysregulation-related neurological disorders and the treatment strategies in these neurological diseases.

## 2. Brain Iron Metabolism

**Figure 1:**
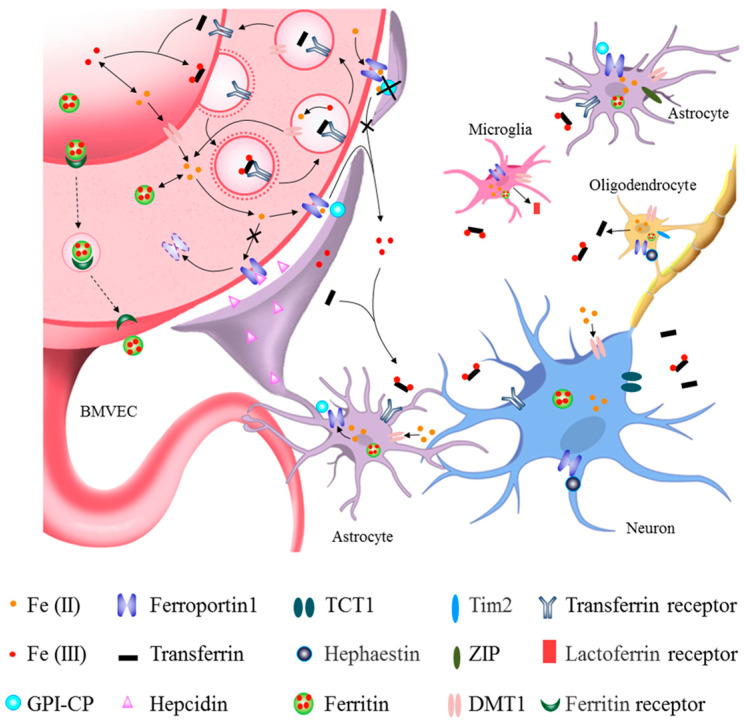
Roles of different cells in brain iron metabolism. The main route of brain iron uptake is where the iron in the blood crosses the blood–brain barrier (BBB) via Tf-TfR1 in the apical surface of brain microvascular epithelial cells (BMVECs) and FPN1 in the basal surface of BMVECs. Iron can also enter the brain through the transcytosis of ferritin by its receptors at BBB. After iron influxes into the brain parenchymal tissue, it can enter astrocytes through their end feet surrounding BBB and then be transferred to neurons. Iron across the BBB can also directly enter the interstitial fluid of the brain and be transferred to neurons and other cells without passing through astrocytes (see black lines). Astrocytes hepcidin secreted through its end feet to directly decrease FPN1 level of BMVECs, which decreased the iron influx into brain tissues. GPI-CP expressed by astrocytes assists FPN1 in releasing iron into the brain. Astrocyte-specific *Cp* knockout blocks iron influx FPN1-CP pathway into the brain (see black lines and crosses). Neurons acquire both trivalent and divalent iron through TfR1, TCT1 and DMT1, while those astrocytes that are not part of the BBB acquire iron via DMT1 and ZIP molecules. Oligodendrocytes mainly uptake iron via DMT1 and Tim2. Oligodendrocytes can secrete Tf, while the activated microglia can secrete Lf. Neurons and glia store iron in ferritin and release iron through FPN1 with the coordination of CP/hephaestin or hepcidin, thereby further promoting cross-talk and interaction with other types of cells.

How iron enters the brain has long been a mystery. Given the lack of evidence of iron release from the brain, iron homeostasis is thought to be primarily maintained via the regulation of iron uptake. The brain acquires iron primarily from the blood and cerebrospinal fluid; uptake across the BBB is thought to be the primary pathway. Recently, the molecular mechanisms of brain iron uptake and transport among neurons and different types of glia have slowly been becoming clear (Figure 1).

### 2.1. Iron Uptake into Brain Parenchyma from Circulatory Iron across the BBB

During infancy, when the BBB has not yet formed, iron is thought to enter the brain directly. In adults with an intact BBB, brain iron uptake mainly occurs in brain microvascular epithelial cells (BMVECs) and depends on transferrin receptor 1 (TfR1) and FPN1 [23,24]. Data have shown that iron in the blood circulation can enter rain parenchymal tissue across the BBB. Transferrin-bound iron in the blood is first taken up by BMVECs by binding TfR1 in the luminal membrane; this is referred to as the Tf-TfR1-dependent iron uptake pathway [23]. However, there is no direct in vivo evidence indicating how iron in BMVECs is released into the brain via the basal surface of these cells. Endothelial cell-specific *Fpn1* knockout mice and conditional knockdown of astrocytic hepcidin mice revealed that endothelial FPN1 acts as a gatekeeper for iron, mediating iron entry into brain tissue from BMVECs [11,25].

The gatekeeper FPN1 is regulated by hepcidin, CP and iron regulatory protein (IRP). Hepcidin is secreted by astrocytes and can decrease the expression of FPN1 in the striatum, cerebral cortex, and hippocampus [12]. Hepcidin can control the entry of iron through FPN1 on BMVECs via a FPN1–hepcidin posttranslational degradation axis [11]. Glycosylphosphatidylinositol-anchored CP (GPI-CP) is mainly expressed in the end feet of astrocytes surrounding the BBB [13,26,27]. Conditional knockout of astrocytic CP caused ID in the brain, providing direct evidence that CP in astrocytes also regulates iron influx into the brain through the BBB [14]. While the level of IRP is negatively regulated by iron levels in the brain, it also regulates *TfR1* and *Fpn1* mRNA via post-transcriptional regulation of the iron-responsive element (IRE)-IRP system [28,29]. These results demonstrate that coordination of Tf-TfR1 and FPN1 plays a critical role in iron efflux into brain parenchyma from BMVECs, and that hepcidin and CP may help maintain iron homeostasis in the brain.

There are other routes of iron entry into the brain [30]. In the non-transferrin-bound iron pathway, which mediates iron entry via BMVECs of the BBB [31,32], ferritin can bind the ferritin receptor or be transcytosed across the BBB [33]. Iron in the cerebrospinal fluid can also enter the brain across the choroid plexus [34,35].

Different brain regions contain different levels of iron, with high iron concentrations in the substantia nigra (SN), red nucleus and globus pallidus [36,37]. It is unclear why such iron heterogenicity exists in the brain. It may be due to differences in iron demand from specific brain nuclei groups or differences in iron absorption, transport or iron release because of the distribution of iron-metabolism-related proteins in different brain regions [38]. Research has shown that iron can be transported along axons from the ventral hippocampus (vHip) to the SN and from the thalamus to the amygdala [39], so it is possible that axonal iron transport may contribute to heterogeneous iron distribution. It is speculated that choroid-plexus-derived transferrin (Tf) in cerebrospinal fluid (CSF) plays a significant role in the export of iron to the blood instead of iron uptake into the brain interstitium because intracerebroventricular injection of [^59^Fe^125^I]Tf did not lead to an observable signal in brain regions distant from the CSF [40]. The glymphatic pathway may also be involved in iron release from the brain [41]. Do the different degrees of iron release result in different levels of iron reduction or overload? Further investigation of iron deficiency or accumulation in different brain regions is warranted.

### 2.2. Iron Uptake and Metabolism in Neurons and Glia

After iron enters the brain parenchyma, it is absorbed by neurons and glial cells for cellular functions. TfR1 and divalent metal transporter 1 (DMT1) are ferric and ferrous iron uptake proteins, respectively; the lactoferrin receptor (LfR) is also responsible for iron uptake. Most brain cells store iron using ferritin and export iron via FPN1 with the assistance of CP and hephaestin (HP) [42,43]. However, different types of brain cells have different dominant iron uptake pathways. Neurons acquire iron through the classical Tf-TfR1-dependent iron uptake pathway and non-transferrin bind iron (NTBI) uptake pathway, such as DMT1 and trivalent cation-specific transporter 1 (TCT1), from brain interstitial fluid [43,44]. Exported iron from neurons is oxidized into the ferric form by astrocytic CP [45]. Astrocytes acquire iron across the interstitial space using NTBI-dependent mechanisms that include citrate, ATP, ascorbic acid, DMT1 and zinc-regulated and iron-regulated transporter-like proteins (ZIP) [46,47]. Astrocytes have also been reported to directly uptake ferrous iron from BMVECs through the end feet [48]. Oligodendrocytes are the only cells that synthesize and release Tf; they acquire iron through DMT1 and the ferritin receptor, T-cell immunoglobulin and mucin domain protein-2 (Tim-2) [49]. Microglia express iron-metabolism-related proteins and are efficient in accumulating iron [50]. Under inflammation, environmental and endogenous stimuli, microglia are activated, resulting in the synthesis and secretion of lactoferrin (Lf), which affects LfR-expressing cells [51,52]. Choroidal epithelia capture iron via TfR1-dependent or TfR1-independent pathways, and they transport iron back to the blood circulation [53]. In the brain, excess iron can be exported back to CSF or interstitial fluid [54].

In addition to strict regulation of iron entry into the brain, iron levels in different brain cell types are also regulated by IRP and hepcidin. When cellular iron levels decrease, the expression of IRP increases [55]. This allows IRP to bind the IRE motif in the 5′ untranslated region (UTR) of *ferritin* and *Fpn1* mRNA and the 3′-UTR of *TfR1* and *DMT1* mRNA, which inhibits translation of ferritin and FPN1 and increases the translation of TfR1 and DMT1. This leads to decreased iron storage and iron efflux, as well as increased iron uptake, which induces elevated cellular iron levels to maintain iron homeostasis in neurons and glia [29,56,57]. Altered levels of iron in the brain also affect the expression of hepcidin in astrocytes, which affects the level of FPN1 through the hepcidin–FPN1 regulatory axis [11,12]. Data from IRP2^−/−^ mice and cell lines have demonstrated that IRP and hepcidin coordinately regulate FPN1 expression [28]. Therefore, coordination and crosstalk between IRE-IRP and hepcidin maintain the dynamic balance of iron levels in the brain.

Expression of CP and hepcidin in astrocytes regulates iron metabolism in BMVECs, neurons and other glia, highlighting the communication between different cells in the brain [11,14]. Astrocytes also affect microglia, which, in turn, affect iron metabolism in neurons [58]. Cell communication and the underlying network are complicated; further investigation will improve our understanding of brain iron metabolism and provide strategies to prevent and cure iron-metabolism-disorder-related neurological diseases.

Erythroferrone (ERFE), a protein newly identified by the Ganz group, may inhibit hepcidin and, thus, regulate iron metabolism in response to erythropoietin stimulation in conditions of stress [59,60]. ERFE has also been detected in the brain using real-time PCR [60], but its role in brain iron metabolism remains unclear. The possibility of other roles of ERFE in the brain will also require further investigation.

While there has been significant progress in our understanding of the mechanisms and regulatory pathways of brain iron metabolism in recent decades, many issues remain to be addressed. Data from synchrotron-based X-ray fluorescence elemental mapping demonstrated that the element that accumulates in the SN is iron and not zinc or copper [61], which may explain why dopaminergic neurons are vulnerable to toxins. Microglia are prone to accumulate iron under conditions of stress. The cross-talk between glia and neurons remains unclear; in particular, how they coordinate to maintain iron homeostasis in physiological conditions and how iron is redistributed and accumulated in pathological diseases will require further investigation. Uncovering a detailed picture of brain iron metabolism and understanding the key mechanisms will have profound preventive and therapeutic potential for neurological diseases.

## 3. Iron, Redox Balance and Oxidative Damage

### 3.1. Iron Dysregulation Induces ROS Generation

Iron dysregulation can be destructive to cells and tissues. Iron overload, especially labile iron in cells and body fluids, can catalyze the conversion of hydrogen peroxide (H_2_O_2_) via the Fenton reaction to generate highly reactive hydroxyl radicals and superoxide anion, leading to the generation of ROS [62,63]. Iron and iron derivatives, such as heme or iron–sulfur clusters, are the essential active centers of many enzymes (e.g., lipoxygenases (LOX), cytochrome P450, NADPH oxidases) involved in ROS generation [64]. Moreover, dysregulation of iron metabolism also induces the generation of reactive nitrogen species, including nitrogen monoxide, dioxide and peroxynitrite, while these radicals can conversely regulate cellular iron homeostasis by modulating the binding affinity of IRP and IRE [65]. The drastic increase in oxidative and/or nitrosative radicals can disrupt the redox balance in the cell and lead to biological damage, a condition called oxidative stress and/or nitrosative stress, which is involved in many diseases and medical conditions [65]. In addition to iron overload, studies have also reported that extremely low levels of iron in cells can also trigger an increase in ROS [8,66]. Under prolonged ID, increased levels of H_2_O_2_ initiate signaling events, resulting in a regulatory loop between H_2_O_2_ and prolonged ID [65]. ID increases superoxide anion levels, resulting in a significant decrease in catalase activity together with rising levels of dehydroascorbic acid, indicating disruption of redox homeostasis, ultimately triggering programmed cell death [67]. Therefore, iron dysregulation is harmful to cells via increasing ROS levels and resulting oxidative damage.

Neural cells are particularly sensitive to ROS assault because of their intense oxidative metabolism, high consumption of oxygen, and propensity to generate high levels of ROS. Iron levels in different regions of the brain increase with aging, making individuals more prone to age-dependent neurodegenerative diseases [68]. For example, high levels of iron are seen in the cerebral cortex and hippocampus of AD patients, as well as in the dopaminergic neurons of the SN of PD patients [69,70]. Iron induces oxidative damage in proteins and lipids, which is involved in many disease processes, such as synaptic dysfunction, neuroinflammation, and neuronal death, and, thus, is considered an important cause of neurodegenerative diseases [71,72,73].

### 3.2. Iron-Induced Neuronal Death

Although ROS are critical for physiological signaling pathways, excess ROS will damage cellular macromolecules, including proteins, lipids and DNA, and the resulting oxidative stress can eventually lead to apoptosis [68,74]. Normally, there are several detoxification systems and antioxidant defense pathways in cells to counter ROS, such as superoxide dismutases, catalases, and glutathione peroxidases (GPx) [75,76]. However, when the generation of ROS drastically exceeds the antioxidant detoxification systems in cells, oxidative stress results in mitochondrial dysfunction, leading to a further increase in ROS formation and Cyt C release [77]. This will trigger the activation of various signaling pathways, such as MAPK, which activates transcription factors, such as the nuclear factor NF-κB, to alter target gene expression, resulting in the upregulation of proapoptotic factors and downregulation of anti-apoptotic factors [64,77]. This, in turn, exaggerates oxidative stress and ultimately leads to programmed cell death.

**Figure 2:**
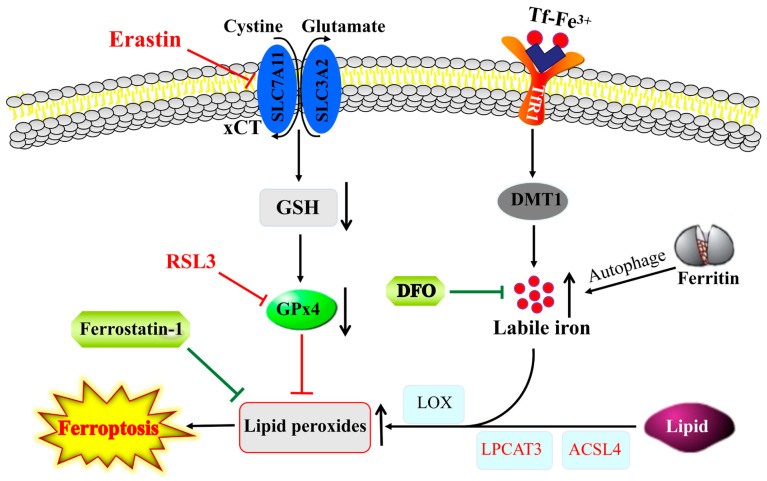
Interplay between ferroptosis and iron homeostasis. Lipid peroxides that induce ferroptosis are produced through auto-oxidation and/or enzymatic activity of LOX on lipid esters generated from lipids via the activity of ACSL4 and lysophosphatidylcholine acyltransferase 3 (LPCAT3). GPx4 blocks ferroptosis by converting lipid peroxides to lipid alcohols, whereas reductions in GSH or GPx4 activity by blocking of xCT antiporter (e.g., by erastin) or inhibiting of GPx4 (e.g., RSL3) can trigger ferroptosis. The increase in labile iron pool in the cytosol via an increased iron uptake through TfR1 and/or autophagic degradation of ferritin can exacerbate ferroptosis via facilitating lipid peroxidation, and, thus, iron chelators, such as DFO and ROS scavengers (e.g., ferrostatin-1), suppress ferroptosis.

Ferroptosis, another form of programmed cell death, is primarily caused by iron-dependent lipid peroxidation (Figure 2). In ferroptosis, upon labile iron accumulation, cytosolic lipid oxidation and ROS are increased, while glutathione (GSH) and GPx4 are decreased, and the mitochondria shrink with an increased membrane density, eventually resulting in cell death [78]. Ferroptosis can be activated by blocking xCT antiporter (e.g., by erastin) or GPx4 inhibitor (e.g., RSL3), while it is inhibited by iron chelators (e.g., DFO) and ROS scavengers (e.g., ferrostatin-1). This process is closely regulated by intracellular signaling pathways, including the iron homeostasis regulatory pathway, RAS pathway and cystine transport pathway [79]. Ferroptosis has been linked to the pathological processes of many diseases, including neurodegenerative diseases of the central system [79,80].

## 4. Role of Iron in Neurological Diseases

**Figure 3:**
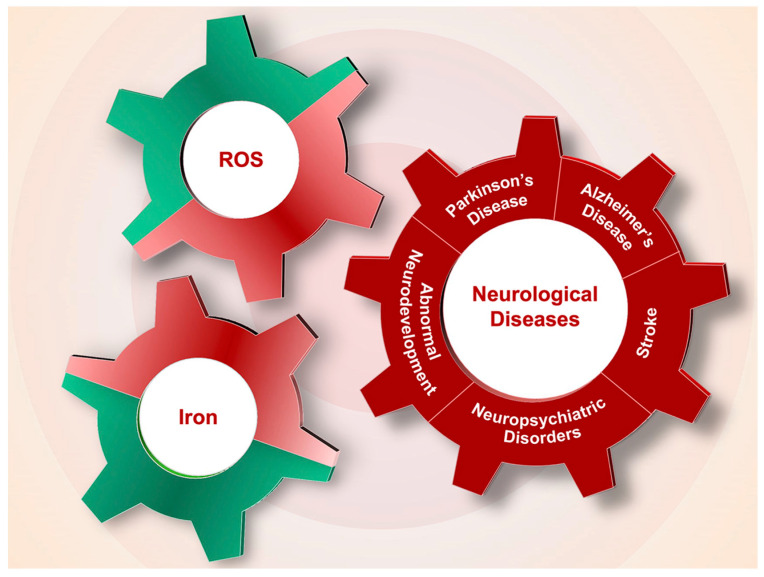
The interplay of iron and ROS in the pathogenesis of neurological diseases. Dysregulation of iron content in neurological system and the associated generation of ROS participate in the pathological processes of Alzheimer’s disease (AD), Parkinson’s disease (PD), stroke, neuropsychiatric disorders and abnormal neurodevelopment.

Iron overload or deficiency is closely related to ROS levels, both of which participate in and exacerbate the onset and progression of neurological diseases, including PD, AD, stroke, neuropsychiatric disorders and abnormal neurodevelopment (Figure 3).

### 4.1. Iron and Parkinson’s Disease

#### 4.1.1. Iron Dysregulation in PD

PD is one of the most typical neurodegenerative diseases, and its discovery was first described by Parkinson in 1817 [81]. PD is characterized by Lewy bodies (LBs) with α-synuclein protein aggregation, as well as death of dopaminergic neurons in the SN and dopamine (DA) deficiency in the striatum [82,83]. While there is significant evidence demonstrating that excess iron accumulates in the SN pars compacta (SNpc) of PD patients and animal models [37,84,85,86,87], it remains unclear whether iron overload is the initial cause or an effect of PD [88]. Although the detailed molecule mechanisms that provoke PD are not fully understood, experimental evidence suggests that iron accumulation occurs earlier [89], and the consequent oxidative damage or mitochondrial dysfunction aggravates the development of PD [90,91]; the evidence also shows that lowering brain iron levels can slow down PD [92,93,94].

CP is a multi-copper ferroxidase that plays important roles in copper transport and converts toxic ferrous into the nontoxic ferric form. CP facilitates iron release from endothelial cells, neurons and glial cells to maintain iron homeostasis in the brain. Aceruloplasminemia is a recessive neurodegeneration characterized by mutation of the CP gene and marked iron accumulation in the brain [95,96,97]. Adult *Cp* gene knockout mice (*CP*^−/−^) show age-dependent iron overload in the central nervous system (CNS) [98,99]; these mice are considered an endogenous iron-overload model. Intracerebroventricular injection of ferric ammonium citrate (FAC), which induces high levels of iron in the brain, is used as an exogenous iron-overload mouse model. Both iron-overload mouse models are more vulnerable to neurotoxin and develop PD following intraperitoneal injection of low-dose 1-methyl-4-phenyl-1,2,3,6-tetrahydropyridine (MPTP). Further investigation demonstrated that iron accumulation is induced by *Cp* gene knockout and exacerbates oxidative stress levels, which promotes apoptosis in the brain. Chelation of iron can decrease brain iron levels and ROS, and apoptosis is reduced in PD mice. Therefore, iron overload in the brain exacerbates dopaminergic neuronal death in the SNpc [90].

Researchers observed a loss of approximately 80% of the ferroxidase activity of CP in the SN of PD cases; *CP*^−/−^ mice developed parkinsonism and exhibited iron accumulation in the SN [100,101]. Thus, *Cp* deletion induced iron overload and consequent ROS elevation, which induced dopaminergic neuron death and led to the onset of PD.

The expression of IRP2, a key regulator of iron homeostasis, is negatively regulated by high levels of iron; altered IRP2 can impact iron uptake, iron storage and iron release proteins through the IRE-IRP regulatory pathway, which maintains iron homeostasis [57,102]. Dominant expression of IRP2 has been detected in the CNS [103]. *IRP2* gene knockout mice (*IRP2*^−/−^), reported by the Rouault lab, develop neurodegenerative movement disorders [104] and exhibit excessive iron accumulation in the brain [56,104,105].

Iron overload is observed in the SN of *IRP2*^−/−^ mice, and low doses of MPTP increase neuronal apoptosis and decrease DA levels by altering iron metabolism, exacerbating parkinsonism symptoms [91]. Levels of the deubiquitylase OTU domain-containing protein 3 (OTUD3) are decreased in PD mice overexpressing A53T α-synuclein. *OTUD3* gene knockout mice showed nigral iron accumulation and dopaminergic neurodegeneration. OTUD3 can stabilize IRP2 to maintain iron homeostasis and prevent PD [106].

Mitochondria ferritin (FtMt) is a protein with ferroxidase activity capable of storing iron in the mitochondria. Although *FtMt* overexpression or deletion does not affect iron levels in the brains of mice, its overexpression induces a slight increase in iron uptake, cytosolic ID and decreases ROS production in SH-SY5Y neuronal cells and significantly blocks iron redistribution in a PD cell model [107]. *FtMt* deletion induced ferroptosis, and its overexpression attenuated ferroptosis during cerebral ischemia/reperfusion [108]. *FtMt* gene knockout promotes ROS generation, and overexpression restricts ROS production in vivo. In vitro, FtMt attenuated oxygen and glucose deprivation and reperfusion-induced iron accumulation in mitochondria [109]. These data revealed that FtMt plays a critical antioxidative role in the progression of PD by regulating ROS.

Nuclear factor erythroid 2-related factor 2 (Nrf2) regulates the expression of antioxidant and detoxification enzymes and participates in many biological functions and disorders including oxeiptosis [110]. *Nrf2* gene knockout decreases the FPN1 level in BMVECs, thus decreasing iron entry into the SN and striatum, reducing ROS and decreasing apoptosis of dopaminergic neurons in PD mice [111], which suggests that Nrf2 is neuroprotective against PD via regulation of iron metabolism in the brain.

In addition to the above mechanisms, other molecules and pathways are involved in iron metabolism disorders in PD. Ferroptosis participates in dopaminergic neuronal cell death in PD, and the mutation of other ferroptosis genes has been linked to PD. On the other hand, an autosomal recessive mutation in the PD-related gene, *DJ-1* (*PARK7*), can suppress ferroptosis [112]. Ferroptosis is also characterized by elevated lipid peroxidation and ROS; therefore, iron and the resulting increase in ROS play prominent roles in the pathology of PD. Overall, there are many reports of iron participation or interaction with neurotransmitters and with many PD gene mutations [113,114,115,116,117,118], which form a vicious cycle that exacerbates the progression of PD.

#### 4.1.2. Iron Overload Exacerbates PD and Related Mechanisms

##### Iron and α-Synuclein

α-synuclein aggregation is thought to play a key role in the formation of LBs that contribute to PD pathogenesis. Iron and α-synuclein both accumulate in LBs of the remaining dopaminergic neurons of the SN [119]. Evidence has shown that iron promotes α-synuclein aggregation [120]; an IRE motif is predicted in the in 5′-UTR of *α-synuclein* mRNA [121], suggesting IRE-IRP mechanisms might regulate its expression. Further investigation showed that iron regulates the synthesis of α-synuclein through the IRE-IRP pathway at the post-transcriptional level; iron-mediated oxidative stress also regulates α-synuclein at the post-translational level. α-synuclein has also been shown to exhibit ferrireductase activity in the SN and may regulate iron uptake [122,123].

Transgenic PD mice overexpressing a mutant (A53T) human α-synuclein exhibited age-related motor deficits, and their SN was more vulnerable to high dietary iron compared with wild-type mice [124]. Excess iron has been linked to increased oxidative/nitrative stress, which could induce tyrosine nitration. Nitrated α-synuclein has been detected in the LBs of the PD brain [125]. The attachment of nitro molecules to Tyr39, Tyr125, Tyr133 and Tyr136 of α-synuclein causes significant changes in α-synuclein [126]. Nitrated α-synuclein is not readily degraded and is mixed into fibrils, accelerating the formation of fibrils with unmodified α-synuclein [127,128,129]. In vitro evidence has demonstrated that microglial activation can induce nitric oxide (NO)-dependent oxidative stress in dopaminergic neurons, resulting in α-synuclein nitration. Nitrated, aggregated α-synuclein during conditions of oxidative stress induces inflammatory microglial functions [130,131].

Phosphorylation of α-synuclein has been shown in LBs [132]. Phosphorylation of Ser129 is the primary modification of α-synuclein [133]; this mutation is harmful in PD. Mutation of S129 (S129D) increases α-synuclein phosphorylation, the aggregation of which promotes dopaminergic neuronal cell death [134,135]. The S129A mutation prevents α-synuclein phosphorylation and suppresses the loss of dopaminergic neurons [134]. While some reports have indicated that α-synuclein phosphorylation has no toxic effects, it remains clear that iron-induced oxidative stress promotes phosphorylation of α-synuclein. Iron overload has been shown to induce phosphorylation of α-synuclein at S129 and its subsequent aggregation in vitro [114,136]. Furthermore, phosphorylation at Y125 or S129 may increase the binding affinity between ferrous iron and the C-terminal region of α-synuclein [115].

Overall, these results suggest that iron and α-synuclein interact with one another, and their deposition and aggregation may be important factors in the pathology of PD. Blocking iron and α-synuclein interactions may be a useful strategy to prevent and cure PD.

##### Iron, Dopamine and Neuromelanin

Decreased levels of the neurotransmitter DA are important in PD. DA generates toxic metabolites in the cytoplasm [137]; iron and DA are considered toxic in combination. Physiologically, DA can produce H_2_O_2_ via monoamine oxidase [113]. H_2_O_2_ produced in dopaminergic enzymatic processes reacts with the high level of iron in dopaminergic neurons, generating oxidative stress such as hydroxyl free radicals via the Fenton reaction. Hydroxyl radicals can damage membrane phospholipids, proteins and nucleic acids to induce neuronal cell death [138].

Iron is also involved in the oxidation of DA, forming 6-OHDA, which liberates iron from ferritin and produces H_2_O_2_, therefore, aggravating dopaminergic neuronal cell death and the development of PD. It was reported that high levels of iron inside cells caused ferroptosis, a form of regulated cell death characterized by iron-dependent accumulation of lipid hydroperoxides to lethal levels [78,139].

Neuromelanin, a dark pigment in dopaminergic neurons, binds reactive iron in neurons and plays a neuroprotective role. Loss of neuromelanin is observed in PD patients [89]. When the binding capacity of neuromelanin for iron is decreased, free iron increases in the SN and induces oxidative damage via the Fenton reaction. Increased iron also reacts with α-synuclein to aggravate oxidative stress and protein aggregation, resulting in neurodegeneration and neuronal cell death. Degenerating neurons also release neuromelanin, which activates microglia, further releasing neuromelanin and initiating neuroinflammation and neurodegeneration [116]. Therefore, iron is involved in DA oxidation by interacting with DA metabolites (such as H_2_O_2_ and neuromelanin) to damage dopaminergic neurons, which accelerates the release of a-synuclein to activate microglia, producing neuroinflammation and participating in the occurrence of PD.

##### Iron and Parkin

Iron can alter Parkin solubility, resulting in its intracellular aggregation. With the depletion of soluble, functional forms of Parkin, proteasomal activity is impaired with cell damage [140]. In 1998, mutation of *Parkin* was identified in autosomal recessive juvenile parkinsonism; iron staining in the SN of these patients was more intense than that of controls and sporadic PD patients [141]. It was hypothesized that iron accumulation might be related to loss of the *Parkin* gene.

More recently, Parkin was reported to be responsible for ubiquitination of DMT1 (+IRE). Expression of 1B-DMT1 isoforms was decreased in SH-SY5Y cells overexpressing Parkin [117]. When fed an iron-supplemented diet, transgenic mice overexpressing DMT1 showed selective accumulation of iron in the SN; expression of Parkin was also upregulated, likely reflecting a neuroprotective response [142]. Expression of DMT1 (+IRE) was also increased in human lymphocytes containing a homozygous deletion of exon 4 of *Parkin* and in the brains of *Parkin* knockout animals. All these data suggested that there might be a feedback interaction between the abnormal iron level with/without aberrant expressions of iron regulatory molecules and the expression and function of Parkin, thus participating in the progression of PD.

##### Iron and Leucine-Rich Repeat Kinase 2

Leucine-rich repeat kinase 2 (LRRK2) is involved in inflammation, autophagy, lysosomal processing and vesicular trafficking [143]. Mutations in the *Lrrk2* gene cause PD and inflammatory diseases [144,145]. Increased iron is present in PD patients with the *Lrrk2* mutation [146]. The underlying mechanisms and their contribution to PD pathology have been investigated. LRRK2 has been shown to activate microglia; mutations in *Lrrk2* can induce cytokine release and inflammation in PD [147,148]. Recent investigations have indicated that LRRK2 can phosphorylate Rab GTPases, regulating vesicle traffic. The RabGTPase Rab8a directly interacts with TfR to aid in TfR recycling to the cell membrane in the iron-uptake pathway [149]. Mutation of *Lrrk2* enhances Rab8a phosphorylation, sequestering Rab8a in lysosomes, resulting in the dysregulation of endolysosomal transport, inhibition of Tf-TfR recycling and enhanced cellular iron accumulation [118,150]. Therefore, LRRK2 may modulate iron metabolism, especially iron uptake and storage in microglia in conditions of neuroinflammation [118].

Excess iron has been observed in dopaminergic neurons of the PD brain. FAC can catalyze the phosphorylation of S935 and S1292 of LRRK2, significantly increasing its activity. Active LRRK2 accelerates ferrous iron uptake, indicating a relationship between iron and LRRK2 in dopaminergic neurons [151].

**Figure 4:**
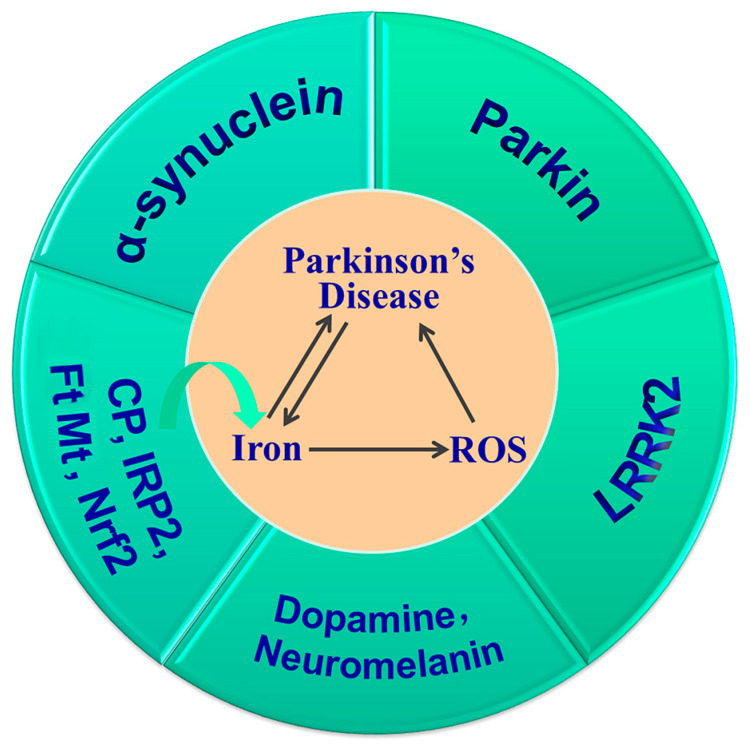
Iron accumulation is important in the pathogenesis of PD. Dysregulation of CP, IRP2, Nrf2 and FtMt alters brain iron levels, which, in turn, affects the expression of iron metabolism proteins. Iron overload and increased ROS aggravate the development and progression of PD, and their interactions with α-synuclein, dopamine, neuromelanin, Parkin and LRRK2 contribute to dopaminergic neuronal cell death and the onset of PD.

In summary, iron deposition in the SN and LB aggregation is a hallmark of PD. The increased iron induced by *Cp*, *IRP2*, *Nrf2* or *FtMt* gene knockout, and the subsequent increased oxidative stress and their interactions with α-synuclein, DA, neuromelanin, Parkin and LRRK2, all contribute to the development and progression of PD (Figure 4). Thus, targeting iron levels is an important strategy in the prevention and treatment of PD.

### 4.2. Iron and Alzheimer’s Disease

AD is a neurodegenerative disease characterized by progressive memory impairment and cognitive dysfunction. The main pathological features of AD are the intercellular deposition of insoluble β-amyloid (Aβ) plaques and intracellular fiber tangles formed by excessive phosphorylation of the tau protein, as well as neuronal cell loss [152]. Although the cause and exact mechanisms of AD have not been revealed, great progress has been made. It has been suggested that the increase in brain iron and the imbalance of iron metabolism associated with increased ROS generation play an important role in the pathogenesis of AD [153,154]. Therapeutic approaches to decrease brain iron levels or restore iron homeostasis along with the attenuation of oxidative stress show great promise in the treatment of AD [154].

#### 4.2.1. Iron Dysregulation in AD

Iron deposition in Aβ plaques and neuronal tangles in the brains of AD patients have been widely reported. In 1992, a study showed that in brain slices of AD patients, the distribution of iron in senile plaques and the surrounding cells increased significantly, suggesting there was iron deposition and disruption of iron homeostasis in the AD brain [155]. Magnetic resonance imaging showed that the aggregation of Aβ accompanied by the accumulation of iron occurred in the early stages of AD [156]. Compared with healthy individuals, AD patients have increased iron in the cerebral cortex and hippocampus, colocalized with the Aβ plaques [156]. The increased levels of iron in the brain exacerbate the aggregation of Aβ and accelerate neuronal cell death. Symptoms of AD can be attenuated by iron-chelating agents [157].

In addition to the changes in iron distribution in the brains of AD patients, the expression of several key molecules responsible for transporting iron or regulating iron homeostasis is also altered, including Tf, TfR1, DMT1, ferritin, FPN1, CP, IRPs and hepcidin [11,14,69,155,158,159,160]. Ferritin in senile plaques in the hippocampus and peripheral blood vessels of AD patients is increased [155,161]; ferritin is also increased in the CSF of AD patients [161]. Protein and mRNA levels of FtMt are increased significantly in the cerebral cortex of AD models [162,163,164]. Expression of TfR1 was shown to be increased in the cerebral cortex and hippocampus of 3-month-old amyloid precursor protein (APP)/PS1 mice [165]. DMT1 in the cerebral cortex and hippocampus of APP/PS1 mice was increased around Aβ plaques [166]. FPN1 in the cortex and hippocampus of AD patients and animal models was significantly decreased [166]. Expression of hepcidin in the brain of AD patients and mouse models was decreased, accompanied by increased neuroinflammation and oxidative damage [58,69,160]. The alterations of these molecules verify the dysregulation of iron metabolism in the pathogenesis of AD.

#### 4.2.2. Iron in Aβ and Tau Pathology

Increased iron directly induces Aβ aggregation, which, in turn, participates in the generation of oxidative stress, contributing to the pathological symptoms of AD [153]. The increased iron can also affect the expression of APP and its subsequent amyloidosis. Under normal conditions, most APP is cleaved by α-secretase and γ-secretase successively, releasing its N-terminal P3 fragment and leaving the APP intracellular domain in the cell membrane, while a small amount of APP undergoes β-secretase (BACE-1) and γ-secretase shearing to produce Aβ [167]. The activation of both α-secretase and BACE1 is regulated by furin, and the transcription of furin is regulated by intracellular iron levels [72,168,169]. When total iron levels are high, furin protein levels decrease [72], which downregulates the activity of α-secretase, resulting in Aβ production. By contrast, in conditions of ID, furin activity increases, increasing α-secretase activity and the stimulation of non-Aβ cleavage of APP [170]. APP translation is also affected by iron levels because of the IRE motif present in the 5′-UTR of APP mRNA, which can be regulated by IRPs upon binding [171]. In conditions of ID, IRPs bind the IRE of *APP* mRNA to inhibit its translation. However, at high iron concentrations, IRPs interact with iron and are released from *APP* mRNA, resulting in increased APP translation, which, in turn, increases Aβ generation [171].

Iron overload can also aggravate tau protein dysfunction and enhance the formation of neuronal fiber tangles. In the brains of AD patients, lipid peroxidation induced by iron overload can promote tau polymerization, which further increases oxidative stress and the formation of tau fibrillary lesions [172]. In vivo experiments have shown that iron overload causes abnormal phosphorylation of tau protein in neurons [173]. Previous studies have shown that a lack of tau can affect the post-translation transport of APP, resulting in its retention in the endoplasmic reticulum [174]. APP exhibits ferrous oxidase activity, which may help iron efflux by stabilizing FPN1 on the cell membrane [175]. Therefore, lack of tau may affect iron release by regulating APP, leading to increased intracellular iron and further aggravating cell damage.

#### 4.2.3. Mechanism of Iron and Oxidative Stress in AD Pathogenesis

**Figure 5:**
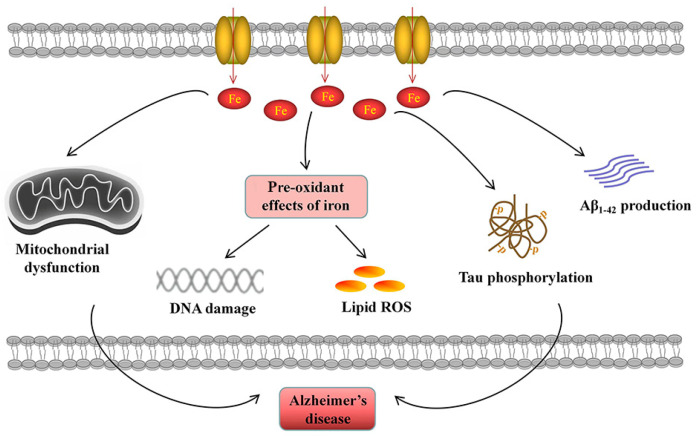
Iron accumulation participates in the pathogenesis of AD. Elevated cellular iron is related to Aβ_1-42_ production and tau phosphorylation. Excessive iron leads to mitochondrial dysfunction. The pre-oxidant effects of iron induce DNA damage and lipid ROS generation, contributing to cell death.

Iron overload and the resulting oxidative stress participate in the pathology of AD symptoms. In AD pathogenesis, iron overload has been implicated in mitochondrial dysfunction, neuroinflammation and neuronal cell death (Figure 5).

Mitochondrial dysfunction is a common pathogenic feature of AD [176], evidenced by elevated ROS generation and lipid peroxidation, decreased mitochondrial membrane potential and altered mitochondrial morphology [176]. Sufficient intracellular iron is required for mitochondria-dependent metabolic activities, such as ATP production by the electron transport chain, Fe-S cluster formation and heme biogenesis. Disruptions in iron homeostasis result in mitochondrial dysfunction and energetic failure. ID impairs mitochondrial metabolism and respiratory activity [8], while iron overload promotes the production of damaging ROS during mitochondrial electron transport [177], which triggers oxidative stress in the brain, resulting in neurological damage and disease development [178,179]. Therefore, iron flux in mitochondria must be precisely regulated.

Neuroinflammation is a characteristic of AD and is mainly mediated by the activation of microglia and astrocytes, which release excess inflammatory factors that result in neuronal impairment [176]. Accumulating evidence has shown a relationship between iron levels and neuroinflammation. Elevated neuroinflammation has been reported to contribute to the deleterious impact of iron overload on brain function in aging through astrocytic dysfunction and inflammation [180]. In AD, iron plays a direct role in Aβ-stimulated neuroinflammation. Iron overload promoted activation of NF-κB signaling induced by Aβ and increased secretion of inflammatory factor interleukin (IL)-1β in microglia [181]. Similarly, iron overload in lipopolysaccharide (LPS)-primed peripheral blood mononuclear cells stimulated caspase 1-dependent IL-1β secretion and activated the NOD-like receptor family pyrin domain-containing 3 inflammasome, due to the increased cellular labile iron [182]. Iron overload has also been shown to promote IL-6 secretion through microglia, which, in turn, upregulates the expression of IRP1 and DMT1 and downregulates the expression of FPN1 via C-Jun N-terminal kinase activation [183], aggravating iron overload and inducing oxidative stress and cellular dysfunction.

On the other hand, iron metabolism is also regulated by inflammatory and anti-inflammatory cytokines, such as tumor necrosis factor (TNF)-α and transforming growth factor (TGF)-β1. TNF-α has been shown to promote iron uptake in astrocytes and microglia by promoting DMT1 expression, while TGF-β1 facilitates iron efflux in astrocytes by increasing FPN1 expression, thereby differentially contributing to iron homeostasis [184]. In addition, hepcidin in astrocytes is important in LPS-induced neuroinflammation and neuronal apoptosis [58]. High hepcidin levels are associated with intracellular iron accumulation, as hepcidin binds FPN1, internalizing the receptor and blocking iron release from cells [28,58,185]. These results suggested that neuroinflammation stimulates iron overload by regulating the expression of iron transporters, followed by a positive feedback loop that aggravates neuroinflammation and oxidative stress in the brains of AD patients. As iron overload and its related chronic neuroinflammation contribute to the progression of AD, iron chelators have been investigated as potential agents to alleviate neuroinflammation and ROS generation [186].

Iron-induced cell death is an important cause of neuronal death in AD pathology. Studies have found that there are ~30- to 50-times more DNA fragments in neurons and glial cells in AD patient brains than in normal brains of humans of the same age [187], indicating that apoptosis is one of the main forms of cell death in the AD brain. Iron overload in AD is accompanied by an increase in apoptotic cells; reducing brain iron levels reduced apoptosis in the cortex and hippocampus of AD mice [69,160]. These results suggest that the iron-induced apoptosis pathway plays an important role in neuronal cell death in AD. In addition to apoptosis, ferroptosis is mainly caused by iron-dependent oxidative damage and is thought to be closely regulated by intracellular iron homeostasis [78]. Iron overload in AD reduces the expression of GPx4 and increases expression of acyl-CoA synthetase long chain family member 4 (ACSL4); restoring iron homeostasis ameliorated AD symptoms by inhibiting ferroptosis [69,79], indicating that iron-accumulation-induced ferroptosis is an important characteristic of AD.

### 4.3. Iron and Stroke

Stroke is the second leading cause of death after cancer, and adults who survive have varying degrees of physical disability. A quarter of people around the world are affected by stroke [188], resulting in a significant burden to society and patients. Ischemic stroke is the most common form of stroke. Due to vascular embolism, continuous occlusion of blood flow leads to irreversible necrosis of nerve cells in ischemic brain tissue, forming the infarction core. Adjacent tissue cells retain some level of metabolic activity, which is referred to as penumbra. At present, the primary treatment is thrombolysis, although reperfusion causes excitoneurotoxicity, Ca^2+^ overload, ROS generation and inflammation, activates innate and adaptive immunity and causes secondary damage to tissues. The mechanism of ischemia/reperfusion (I/R) is complex, and many modes of cell death are involved, including necrosis, apoptosis, autophagy and ferroptosis. Ferroptosis is an important cause of tissue damage and cell death during reperfusion [139]; the use of ferroptosis inhibitors alleviates I/R damage. Our previous study found that brain iron metabolism was disturbed after I/R and iron levels were increased in the cortex and hippocampus. Hepcidin has been shown to regulate iron levels [189] and cells were characterized by brain iron overload. Excess iron can cause lipid peroxidation via the Fenton reaction, which is important in apoptosis [190] and ferroptosis [108]. Therefore, iron plays an important role in neural damage caused by thrombolysis or thrombectomy in ischemic stroke.

#### 4.3.1. Iron Regulation in Ischemic Stroke

Although there have been many reports of iron overload after stroke, the underlying mechanism remains unclear. Previous studies have shown that hepcidin is involved in the regulation of cellular iron overload after stroke [189]. Stroke upregulates hepcidin expression, and hepcidin was shown to bind and internalize to degrade the iron exporter FPN1 [191], which, in turn, leads to iron accumulation in cells. The hypoxic environment created by ischemic stroke also affects intracellular iron and regulates iron-related protein expression, primarily through hypoxia-inducible factor (HIF). Knockdown of *HIF-1α* reduces hypoxia-induced iron accumulation in cells. HIF-1α binds as a transcription factor to the hypoxia-responsive element (HRE) of *Tfr1* and *DMT1*, activating their transcription, which, in turn, increases iron uptake [192]. Glutamate receptors (N-methyl-D-aspartate receptors (NMDARs)) also mediate iron uptake following a stroke. NMDARs induce the Ras protein via NO/dexamethasone, increasing the TfR-dependent iron uptake pathway [193]. In addition to direct the regulation of iron by hypoxia, the BBB is also involved in cerebral blood deposition in I/R. Changes in iron levels in cerebral microvascular endothelial cells can directly affect iron levels in the brain. After oxygen and glucose deprivation and reperfusion (OGD/R) treatment, iron levels in cerebral microvascular endothelial cells were significantly increased [190]. Endothelial cells released intracellular iron into the brain through FPN1, and knockdown of *Fpn1* in endothelial cells decreased iron accumulation in the brain and alleviated oxidative stress, inflammation and cell death after ischemic stroke [25]. The above results suggest that the mechanism of iron metabolism dysregulation in ischemic stroke may not only include the disruption of the BBB and abnormal iron transport across the BBB but also be caused by disorders in iron uptake and release by neural cells in the brain.

#### 4.3.2. Mechanism Underlying Cell Death Induced by Iron Dysregulation

Intracellular iron accumulation after ischemic stroke can aggravate cell death and tissue damage in various ways. Oxidative stress, in particular, is an important factor in excess iron-mediated cell death, including apoptosis and ferroptosis. ROS induces the opening of mitochondrial membrane permeability pores via oxidative damage of lipids and other macromolecules in mitochondria, releasing cytochrome c into the cytoplasm, which activates caspase-3 and triggers apoptosis [194,195]. In the cytoplasm, ROS primarily activates the downstream signaling molecule c-Jun NH2-terminal kinase (JNK) through apoptosis signal-regulating kinase 1 (ASK1) to further phosphorylate the proapoptotic molecule Bak/Bax, resulting in the mitochondrial release of cytochrome c [196,197,198]. In addition to participating in ROS generation and cell death, iron also plays an important role in energy metabolism. Studies have demonstrated that the overexpression of FtMt, on the one hand, decreases the level of free iron in mitochondria and slows the production of ROS after OGD/R. However, FtMt also enhanced glucose metabolism and the pentose phosphate pathway after OGD/R to promote the synthesis of NADPH and glutathione, thus increasing cellular resistance to oxidative damage [109]. Iron also functions as a coenzyme for many oxidases, such as LOX, NADPH oxidase and xanthine oxidase, which are involved in catalytic lipid oxidation and ROS generation [199] and are another important source of oxidative damage.

Based on the role of iron and ferroptosis in I/R, many compounds and therapeutic strategies targeting iron have been explored and validated in animal models. The iron chelator DFO is widely used in a variety of stroke models and has been shown to alleviate stroke injury in mice [200]. Other iron regulatory proteins have been shown to be protective in animal models. Endothelial cells release intracellular iron into the brain through FPN1; while knockdown of *Fpn1* reduces oxidative stress, inflammation and cell death after stroke, ID is not beneficial during neurological recovery after ischemic stroke [25]. Previous studies have shown an increase in iron in the cerebral cortex during post-stroke recovery, along with a corresponding increase in synaptic plasticity and myelin nerve regeneration, indicating that the recovery process, unlike ischemia, seems to require more iron involvement [201]. Knockdown of hepcidin or peripheral injection of CP during ischemia decreased brain iron levels and improved post-stroke motor capacity in mice [189,202]. Intravenous injection of iron-loaded Tf increased cell mortality, increased ROS production and aggravated damage after OGD [203]. Our previous study found that HIF-1α acts as a transcription factor to activate transcription of FtMt, which preferentially sequesters intracellular iron in mitochondria, diminishing free iron in the cytoplasm. Overexpression of FtMt is protective in both hypoxia and I/R models [108,163]. An increasing number of new therapeutic agents targeting iron to treat ischemic stroke are being explored. Nanoliposomes carrying lycopene have been shown to have therapeutic efficacy following ischemic stroke injury in rats. Lycopene nanoliposomes regulate iron levels after stroke and reduce oxidative stress and apoptosis [204]. Moreover, some ferroptosis inhibitors such as Ferrostain-1 were shown to alleviate I/R injury. Further research on brain iron metabolism imbalance following I/R will improve our understanding of the role of iron and provide new directions for prevention and targeted therapies.

### 4.4. Iron and Neuropsychiatric Disorders

Neuropsychiatric disorders, such as depression and anxiety, are increasing worldwide, affecting approximately 30% of the general population during their lifetime [205]. Abnormal synthesis and secretion of neurotransmitters, reduced neuroplasticity and impaired neurodevelopment have been linked to the pathogenesis of neuropsychiatric disorders [206], but the specific mechanisms remain unclear. ID has been correlated with behavioral and developmental changes that occur with neuropsychiatric disorders; these changes affect the hippocampus, the striatum and neurotransmitters, such as serotonin, noradrenaline and DA [206]. Analysis of survey studies found a link between iron intake and depression; total iron intake may be inversely associated with depression [207]. A health survey of individuals over 65 found that a higher number of depressive symptoms was associated with lower hemoglobin levels and higher serum TfR levels but not with ferritin levels [208]. Accumulating evidence demonstrates that ID results in increased anxiety and/or depression with social and attentional problems in children [209]. By contrast, iron overload alters anxiety-like behavior and mood [210]. A recent study confirmed that an imbalance of iron metabolism is a cause of anxiety; researchers found that the neural circuit from the vHip to the medial prefrontal cortex (mPFC) to the SN was responsible for brain iron transport and that dysfunction of vHip-mPFC iron transport could induce anxiety-like behaviors [39]. Monoamine metabolism is the most widely studied metabolic pathway, and iron is required for the synthesis of monoamine neurotransmitters. In particular, serotonin plays an important role in depression, anxiety and other neuropsychiatric disorders [211]. ID results in poor brain myelination and impaired monoamine metabolism, and accumulating data have shown that neurotransmitter homeostasis influences emotional behavior [212].

Fear memories are common in humans and can be used to avoid or minimize harm. Few studies have focused on the role of iron metabolism in the development of fear memory. ID in early brain development can lead to long-term neurological damage, including hippocampus-mediated learning and memory deficits [213]. The hippocampus is an important structure for many forms of memory. The dentate gyrus of the hippocampus plays a key role in the acquisition of situational fear memories [214,215]. In addition, hippocampal-dependent learning was shown to be permanently impaired during fear-conditioning experiments in rats with perinatal ID [216]. An investigation into the response of the mice to contextual fear revealed that the formation of fear memory was impeded after neuronal *Fpn1* depletion by reducing brain iron [7]. Hippocampal-dependent memory processes, such as cognitive memory and fear conditioning, are strongly affected by perinatal ID [217]. These studies suggest that the normal development of the nervous system requires a balance of iron levels in the brain; this balance is important for normal nervous system function and can affect fear memories.

### 4.5. Iron and Abnormal Neurodevelopment

Normal development of the brain is an important process in the establishment of the mammalian nervous system; development involves proliferation, differentiation, migration, synaptogenesis and myelination [218]. During the early stages of mouse embryonic development, neural progenitors divide to give rise to neurons. This is followed by gliogenesis, myelination and synapse construction [219]. Numerous studies have suggested that abnormal brain development is closely related to nervous system disorders, such as microcephaly, schizophrenia, autism spectrum disorder and malformation of cortical development [220,221,222,223,224,225].

Numerous animal studies have shown that iron is particularly important for many neurodevelopmental processes, especially during pregnancy and infancy due to rapid growth [226]. However, ID is one of the most common nutritional deficiencies, especially in pregnant women and infants [209,227]. Numerous studies have shown that ID in these periods causes neurodevelopment deficits, including impairment of learning and memory, motor skills and emotional regulation, and these deficits are not fully recovered even when iron is restored [4,5,6,216,228,229,230,231,232,233,234,235,236,237,238,239]. The mechanisms that may contribute to these impairments include changes in brain energy metabolism, neurotransmitter chemistry, organization and morphology of neuronal networks and the neurobiology of myelination [234,240]. Here, we summarize the major findings from recent decades on the effects of ID on the development and functions of neurons and glia cells (mainly neurons and oligodendrocytes) and highlight new information on the possible mechanisms through which ID affects brain development.

#### 4.5.1. Iron Deficiency Affects Neurodevelopment and Function

Numerous studies have shown that iron is an essential element for cell proliferation by serving as a substrate for enzymes that participate in DNA synthesis, the cell cycle and energy production [241]. Recent studies have revealed the role of iron in maintaining the stemness of embryonic stem cells. Intracellular ID significantly inhibits the proliferation and differentiation of embryonic stem cells/neuronal precursor cells [8,242]. Overall, these data suggest that iron is essential for cell proliferation and differentiation.

Studies in rodents have reported deleterious effects of ID on the structural and morphological development of dendrites and synapses during brain development [243,244,245,246,247]. Furthermore, studies in neurochemistry have shown that ID has significant effects on neuronal DA metabolism [209,233] and the synthesis of monoaminergic neurotransmitters [246,248,249] and growth factors [243]. ID also leads to changes in the synaptic transmission and synaptic function [250,251]. A study using two non-anemic genetic ID mouse models (knockout of *DMT1* or overexpression of dominant negative *TfR1*) showed that neuronal-specific ID dysregulates mammalian target of rapamycin (mTOR) signaling during hippocampal development [252]. Together, this research indicates that ID alters the neuronal structure and morphology, metabolism, synaptic plasticity and structural gene expression and mTOR signaling pathway.

#### 4.5.2. Iron Deficiency Affects Development of Oligodendrocytes and Myelination

Oligodendrocytes are characterized by high intracellular iron and a high rate of oxidative metabolism, which are required for the synthesis and maintenance of myelin [6,253,254]. Studies in mice have also shown that ID negatively affects the development of oligodendrocytes and their myelination [244,255,256,257]. Impaired myelination has also been reported in studies of gestational and postnatal ID performed in monkeys and piglets, suggesting that iron is essential for oligodendrocyte activity and integrity [258,259].

#### 4.5.3. Mechanisms Underlying ID and Hippocampal Development

Based on the vulnerability of the developing hippocampus to early ID and earlier work showing lasting spatial memory deficits related to the role of the hippocampus in iron-deficient rodent brains [216,260,261,262], more attention has been paid to ID and hippocampal function. ID during late fetal and early postnatal life alters the expression of critical genes involved in hippocampal development and function, including iron metabolism, cell growth, energy metabolism, dendrite morphogenesis and synaptic connectivity in the hippocampus [213,243,252,263,264,265,266]. DNA methylation and O-linked-beta-D-N-acetylglucosamine (O-GlcNAc) modifications play important roles in these processes [267,268]. Recently, ID response networks and signatures have been revealed through quantitative proteome and transcriptome dynamics analysis in neuronal cells [269]. Taken together, these studies suggest that ID induces changes at the proteome and transcriptome levels, as well as alterations in post-translational modifications, including phosphorylation signaling and DNA methylation.

Long-term studies show that many of the ID-induced neurodevelopmental deficits during the fetal and early postnatal period cannot be recovered by iron repletion at later stages and eventually lead to sustained impairments [259,270,271,272,273], suggesting that ID in the developmental stage results in long-lasting abnormalities, even after iron supplementation. At present, studies support the concept that early ID in critical periods may disrupt the timing of key steps in development, and repletion of iron after these important time points will not rectify anatomic and neurochemical abnormalities. While awaiting prospective trials, it is recommended to screen all gravidas for ID and administer iron supplementation if ID is present with or without anemia.

In conclusion, numerous studies in human and animal models suggest that ID affects brain development and significantly affects the development and function of neurons and oligodendrocytes. At present, the possible mechanisms of ID affecting brain development are derived from studies on animal models of deficits in brain energy metabolism, neurotransmission, neuronal morphology and the myelination of oligodendrocytes. Since ID in pregnant women and pre-school-age children causes poor long-term neurodevelopment outcomes in later life, it is of important scientific, medical and social significance to further clarify the molecular mechanisms of ID affecting brain development, as well as prevention and treatment strategies.

## 5. Targeting Iron Metabolism in the Treatment of Neurological Diseases

In recent years, research on brain iron metabolism disorders and neurological diseases has shown that increases in brain iron and imbalances in iron metabolism may play essential roles in the pathogenesis of neurological diseases. Therefore, targeting brain iron homeostasis and regulation of iron-metabolism-related molecules for drug development are expected to provide novel ways to treat neurological diseases.

### 5.1. Iron Chelators

Iron chelators bind iron ions with high affinity, effectively enhancing iron excretion and reducing free iron in the body [274]. Iron chelators have been shown to inhibit lipid peroxidation and reduce ROS levels in neurons, thereby preventing neuronal ferroptosis and apoptosis. Clinically, commonly used iron chelators include DFO, DFX and DFP, which have shown therapeutic promise in preclinical and clinical models of neurological disorders. Decreasing iron levels in the brain with iron chelators has been reported to alleviate the symptoms of AD, PD and stroke [17,18,19,20,21,22].

#### 5.1.1. Iron Chelators in AD

Clinical application of DFO to treat AD was reported as early as 1991; continuous administration of DFO was found to alleviate cognitive impairment in AD patients [17]. Administration of the iron chelator M30 decreased brain iron accumulation, Aβ accumulation and tau phosphorylation, improving memory deficits in APP/PS1 mice [18]. This improvement may have been achieved by downregulating phosphorylation of cyclin-dependent kinase 5 (CDK-5) and increasing phosphorylation of protein kinase-B (PKB/AKT) and glycogen synthase kinase (GSK)-3β [18]. DFO improved the cognitive ability of APP/PS1 mice due to the activation of M2-type microglia and inhibition of activation of M1-type microglia in the hippocampus [275].

#### 5.1.2. Iron Chelators in PD

Overloaded intracellular iron contributes to neuronal cell death in PD via apoptosis and ferroptosis, while DFO can inhibit ferroptosis to protect neurons [19]. In experimental studies, iron chelators have been shown to exhibit neuroprotective effects in vivo against 6-OHDA-induced neurotoxicity in mouse models of PD [20]. Loss of CP iron oxidase activity in the SN of PD patients leads to the accumulation of iron peroxide [100]. Administration of an iron chelator in CP^−/−^ PD mice reversed the accumulation of iron ions caused by the loss of CP, significantly improved the motor ability of mice and reduced the nerve damage caused by MPTP [90,100]. After 8 weeks of pre-administration of the iron chelator clioquinol in a mouse PD model, iron levels in the SN decreased by 30%, and oxidative stress and GSH loss were significantly reduced [94]. Treatment with the iron chelator DFO has been shown to block MPP^+^-mediated damage of dopaminergic neurons and prevent iron accumulation and mitochondria dysfunction [276]. Brain iron accumulation exacerbates the pathogenesis of MPTP-induced PD; DFO alleviates PD symptoms by reducing oxidative stress damage caused by elevated brain iron levels [90]. In two phase 2 trials, the high-affinity iron chelator DFO was shown to reduce iron accumulation and improve motor symptoms in PD patients compared with placebo, despite the side effects, such as leukopenia, gastrointestinal discomfort and joint pain [277,278]. A clinical trial evaluating the effects of four different doses of DFP on 140 patients with early-stage PD has yet to be published [278]. A more extensive European multicenter test on the protective effect of DFP on PD patients showed that the iron content in the nigrostriatal pathway was significantly reduced in DFP-treated groups. The mean change in the total movement disorder society-sponsored revision of the unified Parkinson’s disease rating scale (MDS-UPDRS) score was 16.7 points in the DFP group and 6.3 points in the placebo group [279]. Surprisingly, DFP without DA treatment increased the patient’s disability. This may be related to the fact that iron is a cofactor in DA synthesis. Iron is required to assist in the synthesis of DA, but excess iron can cause the death of dopaminergic neurons [278]. Another long-term clinical trial used an iron chelator combined with DA to avoid the drawbacks of iron chelators alone, but further research is needed.

#### 5.1.3. Iron Chelators in Stroke

Iron-overloaded animals are more affected by middle cerebral artery occlusion [280], whereas iron chelation or depletion reduces I/R-induced brain injury [21,22]. DFO has been shown to inhibit lipid peroxidation and hydroxyl radical production via the Fenton reaction and to decrease cerebral I/R-associated brain injury [281,282]. DFO decreases excitatory amino acid levels and improves the histological outcome in the hippocampus of neonatal rats after hypoxia–ischemia [283]. Gerbils fed a low-iron diet for 8 weeks had decreased brain iron levels, neurologic deficits and brain edema after cerebral I/R [22]. Treatment with DFO resulted in decreased brain edema following I/R [22]. DFO treatment attenuated oxidative damage and cell loss induced by oxygen–glucose deprivation followed by reoxygenation in a cell model of cerebral I/R [108,190]. Most stroke-related clinical trials have focused on the treatment of intracerebral hemorrhage. Thus, 294 participants with intracranial hemorrhage were recruited to participate in the safety and efficacy evaluation of DFO and placebo, which showed that deferoxamine mesylate was safe, and DFO treatment significantly improved clinical outcomes [284]. In two clinical trials, DFO treatment appeared to accelerate recovery [285] and reduce hematoma volume [286] in patients with cerebral hemorrhage.

In conclusion, iron chelators are commonly used to reduce the level of iron in the brain, which attenuate oxidative damage, inhibit neuronal ferroptosis and apoptosis and effectively relieve the symptoms of AD, PD and stroke. However, the clinical application of iron chelators still needs to better explore drug administration to improve the therapeutic effect.

### 5.2. Iron Chelators in New Administration Forms

DFO, a hydrophilic drug that binds extracellular iron in a ratio of 1:1, has low oral availability, poor BBB permeability and a short half-life [287]. By contrast, DFX and DFP have higher oral bioavailability and intracellular iron affinity [287,288]. The main advantage of DFP is that it can cross the BBB and chelate iron in cells in the brain [288]. However, the binding ratio of DFP to iron is 3:1, which is lower than that of DFX to iron (2:1), so DFP is less likely to consume stored iron in the body [287,289]. DFP tends to mechanically cross the cell membrane, form complexes with iron, leave the cell and redistribute iron to Tf for recycling [290,291]. Therefore, the use of iron chelators alone in the treatment of neurological diseases is limited.

New forms of administration are being developed to reduce the side effects of DFO and improve its ability to penetrate the BBB. A substantial body of preclinical evidence and early clinical data has demonstrated that intranasal delivery of DFO and other iron chelators has strong disease-modifying impacts in AD, PD and ischemic stroke [292]. Administration of DFO to APP/PS1 mice through the nasal cavity significantly diminished iron-induced tau phosphorylation, APP expression and Aβ accumulation, improving the cognitive decline in mice [293,294]. This result may be due to the inhibitory effect of DFO on iron-induced tau phosphorylation through CDK5 and GSK-3β pathways [293], as well as upregulation of HIF-1α expression via activation of the MAPK/P38 pathway and HIF-1α-mediated regulation of iron metabolism [294]. This, then, ultimately decreased iron levels in the CA3 region of the hippocampus [294]. Intranasal drug delivery allows for direct targeting of drugs to the brain, bypassing the BBB and minimizing systemic adverse effects [295,296]. Improvement in motor deficits and dopaminergic neuronal survival with non-invasive intranasal delivery of DFO in 6-OHDA-induced PD has been reported [296]. Intranasal administration of DFO decreased pathological α-synuclein formation at the terminal level and slowed PD progression [292]. Intranasal administration targets DFO to the brain and reduces systemic exposure; intranasal DFO has also been shown to prevent and treat stroke damage after middle cerebral artery occlusion in rats [297].

Nanodrug delivery systems are also being used to increase the efficiencies of drugs such as iron chelators in the brain to treat neurological diseases [298]. Targeted brain delivery of rabies virus glycoprotein 29-modified DFO-loaded nanoparticles developed by our team, which can cross the BBB through receptor-mediated endocytosis, significantly increased entry of DFO into the brain and prolonged the half-life of DFO [92]. Administration of these nanoparticles significantly decreased iron content and oxidative stress levels in the SN and striatum of PD mice and effectively reduced dopaminergic neuron damage and reversed neurobehavioral deficits, without causing any overt adverse effects in the brain or other organs [92]. DFO-loaded nanoparticles are also being investigated to target decreased brain iron levels in AD and ischemic stroke.

### 5.3. Key Molecules of Brain Iron Metabolism as Targets

Studies have found that long-term use of iron chelators can cause side effects [279]. An increasing number of studies have been conducted on the regulation of key molecules of iron metabolism in the brain as therapeutic targets. Experiments in SH-SY5Y cells stably overexpressing the human APP Swedish mutation revealed that decreasing expression of the iron intake protein DMT1 can decrease iron flow into cells and, thus, reduce Aβ secretion [299]. FtMt overexpression can restore Aβ-induced changes in iron and iron-metabolism-related proteins and has a neuroprotective effect on Aβ-induced neurotoxicity [164]. Specifically, increasing the level of FtMt in the brain may be a novel strategy to prevent or treat AD. CP overexpression in the brain of mice via injection of a *Cp* gene plasmid into the lateral ventricle diminished brain iron and hippocampal cell apoptosis, reducing Aβ-induced memory dysfunction in mice [159], providing a theoretical basis for the development of CP as an effective treatment for AD. Conditional knockout of astrocyte *Cp* significantly decreased brain iron levels; iron was deposited in BMVECs, resulting in diminished iron levels in neurons and glial cells [14]. In terms of alleviating iron deposition in the brains of elderly mice, astrocyte *Cp* knockout reduced tau phosphorylation and Aβ deposition and alleviated ROS-MAPK-pathway-mediated apoptosis, thus improving cognitive function [14]. Overexpression of hepcidin in astrocytes downregulated FPN1 in BMVECs, inhibited iron entry into the brain [11], decreased iron levels in the brain and neurons of APP/PS1 mice and reduced oxidative stress and neuroinflammation, ultimately reducing neuronal cell death of APP/PS1 mice and alleviating the symptoms of AD [69,160]. Finally, overexpression of hepcidin in astrocytes delayed the pathological process of AD and effectively improved the spatial cognitive ability of aged mice [69,160].

Overexpression of ferritin in dopaminergic neurons significantly decreased iron levels in the SN and alleviated oxidative stress damage in dopaminergic neurons in MPTP-induced PD models. Overexpression of ferritin heavy chain (FTH1) inhibits ferroptosis and mitochondrial dysfunction in the 6-OHDA model of PD through decreased iron accumulation and ferritinophagy [300]. *Nrf2* knockout prevented entry of iron into the brain, reduced ROS levels and apoptosis of dopaminergic neurons in the SN and improved the exercise ability of elderly mice [111]. FtMt has been shown to inhibit erastin-induced ferroptosis by regulating iron homeostasis and reducing lipid peroxidation levels [301]. Overexpression of FtMt inhibits mitochondrial damage, decreases ROS generation and lipid peroxidation and alleviates 6-OHDA-induced neuronal damage [107]. Overexpression of FtMt suppresses MPTP-induced cell damage in PD by regulating iron metabolism and attenuating oxidative stress [302].

Overexpression of FtMt attenuates cerebral I/R injury by inhibiting iron-mediated ferroptosis [108]. Overexpression of FtMt enhances BBB integrity following ischemic stroke in mice by maintaining iron homeostasis in endothelial cells [190]. FtMt alleviates apoptosis by enhancing mitochondrial bioenergetics and stimulating glucose metabolism in cerebral I/R [109]. Knockout of *Fpn1* in BMVECs can significantly reduce the injury caused by acute cerebral ischemia; the underlying mechanism has been linked to a reduction in iron, oxidative stress and the inflammatory response and a reduction in iron-mediated cell death and apoptosis [25]. Regulating the expressions of critical molecules in iron metabolism, such as FtMt, CP and hepcidin, can effectively restore the brain iron homeostasis, reduce ROS and, thus, alleviate the symptoms of AD, PD and stroke. Regulation of the expressions of these critical molecules in brain iron metabolism is expected to be a potential new therapeutic strategy for these diseases.

### 5.4. Iron Supplements

ID affects neurotransmitter homeostasis and neurodevelopment and has been linked to the pathogenesis of neuropsychiatric diseases. The use of iron supplements is expected to play a positive role in these diseases. Intranasal administration of nanoliposome-encapsulated FAC successfully increased brain iron content [303]. ID reduces cortical plasticity and delays neurological recovery after ischemic stroke [201]. The use of iron supplements can promote endogenous repair in ischemic stroke [201]. *IRP* knockout decreased iron levels in embryonic stem cells and inhibited stem cell proliferation and differentiation by increasing ROS production and decreasing iron–sulfur cluster proteins [8]. With iron supplements, stem cells differentiated normally [8].

### 5.5. Antioxidants and Anti-Inflammatory Reagents Regulate Iron Metabolism as Targets

In addition to chelation of excess iron with iron chelators and maintenance of brain iron homeostasis by regulating key molecules in brain iron metabolism, antioxidants and anti-inflammatory reagents can also regulate iron metabolism and influence lipid peroxidation and neuroinflammation, thus showing great potential in the treatment of different neurological diseases.

Vitamin E treatment can reduce oxidative stress and lipid peroxidation, improve mitochondrial function, attenuate intracellular iron accumulation and recover cell morphology of fibroblasts in PLA2G6-associated neurodegeneration [304]. Melatonin is a free radical scavenger and has the property of iron chelating, which can effectively inhibit iron-overload-mediated oxidative stress and ameliorate oxidation/nitrosation injuries [305]. The iron levels, oxidative stress markers and inflammatory markers were determined and compared in 40 PD patients and 46 controls. It was found that while the iron level was disturbed in PD patients, the content of their antioxidants, such as plasma vitamin C, was lower, and the oxidative stress and the inflammation levels were increased [306]. This indicates that the low level of antioxidants is corrected with the production of free radicals, leading to the neurodegeneration in PD [306]. On the contrary, increasing levels of the antioxidant vitamin C may help improve neurological conditions. Coenzyme Q10 (CoQ10) is a lipophilic antioxidant that can reduce lipid peroxidation levels [307]. Ferroptosis suppressor protein 1 (FSP1) can catalyze the CoQ10 reduction to ubiquinol by NADPH, restoring the antioxidative effects of CoQ10 [308]. The FSP1/CoQ10 pathway prevents irreversible ferroptosis by reducing lipid peroxides [278]. A multicenter RCT was reported to increase CoQ10 activity and slow the functional decline in PD [278,309]. Subsequent larger clinical studies have shown that the treatment effect of CoQ10 in PD patients is not obvious [278]. Therefore, optimizing the dosages and combinations of antioxidants and considering the potential interactions with other treatments are needed in developing antioxidants as therapeutic strategies.

Tea flavonoids (catechins) have been reported to possess the activities of divalent metal chelating, antioxidant and anti-inflammatory, with the advantage of penetration of BBB [310], showing protective effects in different neurological diseases [311]. The bioactive components of green tea, red wine, arctic root and dwarf periwinkle have been shown to have neuroprotective, antioxidant, anti-inflammatory and iron-chelating potential. They may treat neurological diseases at the cellular level by decreasing microglia activation, attenuating damage from ROS, chelating iron and promoting cell growth [312].

To date, most of the research on different neurological diseases focuses on the manifestations and pathogenesis of a single disease to study the treatment strategies. Drugs on the market and in development also tend to target a single neurological disorder or symptom, lacking the ability to explore the common causes of different neurological diseases. The studies have shown that oxidative stress injury and ferroptosis of neurons caused by dysregulation of brain iron metabolism are common issues in the occurrence and development of different neurological diseases. Therefore, targeting brain iron metabolism and designing drugs or therapeutic strategies for the common etiology of different neurological diseases may reduce or inhibit the occurrence and development of these neurological diseases at the source. However, targeting brain iron metabolism to treat these diseases may also have shortcomings and face certain challenges. The insufficient targeting of iron chelators to the brain may affect peripheral iron metabolism, leading to disorder in the systemic iron metabolism and damage to peripheral organs. Moreover, the currently identified targets that can regulate iron metabolism do not exhibit brain-specific expression patterns, and most of them are still in the laboratory stage, lacking clinical data. Thus, further explorations are needed to accurately target specific brain regions and improve delivery efficiency. Therefore, further exploration is needed to accurately target specific brain regions and improve delivery efficiency in the development of drugs that regulate brain iron metabolism.

## 6. Conclusions and Prospects

In this review, we have summarized and elucidated the interplay between dysregulation of iron metabolism, redox imbalance and different neurological diseases. We focused on the mechanisms of iron-induced oxidative damage in disease pathogenesis and proposed the broad application of targeting the regulation of brain iron metabolism to treat neurological diseases. However, the current research faces certain challenges. The mechanism of iron release from brain tissue is unknown, the specific iron metabolism pathways in different nerve cells remain unclear and the role of oxidative stress in the induction of neural damage is not fully understood. Furthermore, translational studies and clinical trials on the optimal use of iron chelators and regulators in targeting iron metabolism in neurological disease are relatively few. Untangling these issues in the future will aid in our ability to better target the regulation of brain iron metabolism for the prevention and treatment of neurological diseases.

## Data Availability

Data is contained within the article.
